# miR-181d-5p ameliorates hypercholesterolemia by targeting *PCSK9*

**DOI:** 10.1530/JOE-23-0402

**Published:** 2024-07-29

**Authors:** Yu Wang, Fan Li, Xiaoqian Gao, Huahui Yu, Zhiyong Du, Linyi Li, Yunhui Du, Chaowei Hu, Yanwen Qin

**Affiliations:** 1 Beijing Anzhen Hospital, Capital Medical University, Beijing Institute of Heart Lung and Blood Vessel Disease, Beijing, China; 2 The Key Laboratory of Remodeling-Related Cardiovascular Diseases, Ministry of Education, Beijing Anzhen Hospital, Capital Medical University, Beijing, China; 3 National Clinical Research Center for Cardiovascular Diseases, Beijing Anzhen Hospital, Capital Medical University, Beijing, China

**Keywords:** hypercholesterolemia, LDL-C, miR-181d-5p, PCSK9

## Abstract

Hypercholesterolemia is an independent risk factor for cardiovascular disease and lowering circulating levels of low-density lipoprotein cholesterol (LDL-C) can prevent and reduce cardiovascular events. MicroRNA-181d (miR-181d) can reduce the levels of triglycerides and cholesterol esters in cells. However, it is not known whether miR-181d-5p can lower levels of circulating LDL-C. Here, we generated two animal models of hypercholesterolemia to analyze the potential relationship between miR-181d-5p and LDL-C. In hypercholesterolemia model mice, adeno-associated virus (AAV)-mediated liver-directed overexpression of miR-181d-5p decreased the serum levels of cholesterol and LDL-C and the levels of cholesterol and triglyceride in the liver compared with control mice. Target Scan 8.0 indicated Proprotein convertase subtilisin/kexin type 9 (*PCSK9*) to be a possible target gene of miR-181d-5p, which was confirmed by *in vitro* experiments. miR-181d-5p could directly interact with both the *PCSK9* 3′-UTR and promoter to inhibit *PCSK9* translation and transcription. Furthermore, Dil-LDL uptake assays in *PCSK9* knockdown Huh7 cells demonstrated that miR-181d-5p promotion of LDL-C absorption was dependent on PCSK9. Collectively, our findings show that miR-181d-5p targets the *PCSK9* 3′-UTR to inhibit *PCSK9* expression and to reduce serum LDL-C. miR-181d-5p is therefore a new therapeutic target for the development of anti-hypercholesterolemia drugs.

## Introduction

Hypercholesterolemia is an independent risk factor for cardiovascular disease (Trinder *et al.* 2020). The global burden of dyslipidemia has increased over the past 30 years (Pirillo *et al.* 2021). Evidence from genetic studies and prospective epidemiological cohort studies has established LDL as a causal factor in the pathophysiology of atherosclerotic cardiovascular disease (ASCVD) (Ference *et al.* 2017). Therefore, lowering LDL cholesterol levels is a strategy for the prevention and treatment of ASCVD (Mach *et al.* 2020). Current pharmacological options for lowering low-density lipoprotein cholesterol (LDL-C) include statins or ezetimibe, which inhibit cholesterol synthesis and absorption, respectively, and alirocumab/evolocumab, which promote LDL-C clearance. Statins have some specific adverse side effects on muscle and glucose hemostasis, and increase the risk of hemorrhagic stroke (Mach *et al.* 2018). Alirocumab/evolocumab are expensive and have the side effects of itchy injection sites and flu-like symptoms in some patients. A greater understanding of the mechanisms regulating LDL-C is therefore needed for the development of new drugs. Despite wide prescription drug use, a large portion of patients experience recurrent cardiovascular disease, and their LDL cholesterol levels fail to reach the target levels recommended in the guidelines (Wang *et al.* 2022).

MicroRNAs (miRNAs) regulate multiple stages of cholesterol metabolism and can be targeted with drugs to lower cholesterol (Citrin *et al.* 2021). Therapeutic manipulation of relevant miRNAs through miRNA mimics or inhibitors may be a practical approach to reducing elevated LDL-C levels in patients with hypercholesterolemia and may lower the risk of cardiovascular disease events (Citrin *et al.* 2021). Clinical trials have been conducted for miRNA-based therapies targeting miR-34 and miR-122 (Janssen *et al.* 2013, Zhang *et al.* 2019).

miR-181d potently reduces the abundance of liver fat droplets by approximately 60% and reduces levels of cell triglycerides and cholesterol esters (Whittaker *et al.* 2010). Furthermore, miR-181d levels are decreased in the plasma and adipose tissue of obese people (Abu-Farha *et al.* 2019). However, no relationship between miR-181d and LDL-C has been reported.

Here, we found a reduced expression of miR-181d-5p through siRNA omics analysis of high-cholesterol mouse and hamster models. We found that miR-181d-5p can reduce the increase in plasma LDL-C induced by a high-cholesterol diet in mice. We also suggest a potential molecular mechanism for the effect of miR-181d-5p on LDL-C through the regulation of proprotein convertase subtilisin/kexin type 9 (*PCSK9*).

## Materials and methods

### Animal experiments

All animal experiments complied with the Institutional Animal Care and Use Committee of Capital Medical University (Beijing, China). The animal research was approved by the Animal Protection and Utilization Institutional Committee of Capital Medical University. The mice were housed at 27°C in a light–darkness cycle controlled environment, with free access to water and standard mouse food or high-cholesterol food.

#### Hypercholesterolemia mouse model

Eight-week-old male C57BL/6J mice were purchased from Beijing Weishanglide Boitecknowledge Co., Ltd (Beijing, China). Normal chow diet (chow) mice were fed a regular diet and water (*n* = 6). The high-cholesterol diet (HCD) mice were fed a high-cholesterol diet (D12108C, 1.25% cholesterol) for 2 weeks (*n* = 6), during which their food intake was recorded. At the end of this period, all mice were euthanized and their body and liver masses were recorded. Liver tissues were dissected, cut into small pieces, and stored at −80°C.

#### Hypercholesterolemia hamster model

Six-week-old male Syrian golden hamsters were purchased from Beijing Weitonglihua Experimental Animal Technology Co., Ltd. Normal control group (chow) hamsters were fed normal feed and water. HCD hamsters were fed a high-cholesterol diet (Cat# C11953, 0.5% cholesterol, 23% fat) for 4 weeks. The hamsters were then euthanized after fasting for 4 h, and livers and sera were collected.

#### miR-181d-5p-overexpressing HCD mouse model

Eight-week-old male C57BL/6J mice were purchased from Beijing Weishang Lide Biotechnology Co., Ltd. After 2 weeks of feeding on a high-cholesterol diet (D12108C, 1.25% cholesterol), mice were injected with 200 µL of AAV at a concentration of 1×10^12^ titers/mL (μg/mL) through the tail vein. The control group (*n* = 6) was injected with the same amount of control virus. Mice in both groups continued to be fed the HCD for 2 weeks and were then killed after fasting for 4 h. Livers and sera were collected for subsequent analysis.

### Cell culture

Huh7 cells were cultured in Dulbecco’s modified Eagle’s medium (Gibco) containing 10% fetal bovine serum, 2 mM l-glutamine, penicillin 100 μg/mL, and streptomycin sulfate 100 μg/mL in a 5% CO_2_ incubator at 37°C. 293T cells were cultured in Dulbecco’s modified Eagle’s medium (Gibco) containing 10% fetal bovine serum (Gibco) and 1% antifungal antibiotic (Thermo Fisher Scientific) in a 5% CO_2_ incubator at 37°C.

Transfection of miR mimics and anti-miRs. The miR mimics and anti-miRs were purchased from Shanghai Genechem Co., Ltd. Huh7 cells at 50%–70% confluency were transfected with 10 µL pre-miR or anti-miR using HiTransG A (Shanghai Genechem Co., Ltd.) in Opti-MEM medium for 16 h. The medium was then changed to a complete growth medium. At 48 h after transfection, cells were lysed. Equal amounts of miRNA Mimic Negative Control #1 (Pre-Ctrl) or anti-miR miRNA Inhibitor Negative Control #1 (Anti-Ctrl) were used as controls for miR mimic or anti-miR experiments, respectively.

### Western blot analysis

Protein samples were extracted from mouse liver tissue lysates and Huh7 cells. In total, 20 mg samples of liver tissue were lysed in RIPA buffer (Solarbio, Beijing, China) by ultrasonication. After centrifugation at 13,400 ***g*** for 20 min, the supernatants were collected, and the protein content was analyzed by western blotting. Proteins were separated by sodium dodecyl sulfate-polyacrylamide gel electrophoresis and then transferred onto polyvinylidene difluoride membranes. The membranes were blocked using 5% skimmed milk and then sequentially incubated with appropriate primary and secondary antibodies. The primary antibodies used were an anti-LDLR polyclonal antibody and an anti-PCSK9 polyclonal antibody (all from Cohesion Biosciences, London, UK).

### Real-time quantitative PCR

Real-time polymerase chain reactions were performed to quantify specific mRNAs. RNA was isolated from liver samples using Trizol (Life Technologies, Thermo Fisher Scientific) and converted to cDNA using a reverse transcription kit purchased from Thermo Fisher Scientific. RT-PCR was performed using the SYBR green method on a real-time PCR system. The relative abundance of each mRNA species was evaluated using the 2^−ΔΔCT^ method. The primer sequences used were as follows: LDLR, forward 5′-CTGTAGGGGTCTTTACGTGTTC-3′, reverse 5′-GTTTTCCTCGTCAGATTTGTCC-3′; PCSK9, forward 5′-AGCAGCCAGGTGGAGGTGTATC-3′, reverse 5′ -CTTGCTCGCCTGTCTGTGGAAG-3′; GAPDH, forward 5′-GGTGAAGGTCGGAGTCAACG-3′, reverse 5′-CAAAGTTGTCATGGATGHACC-3′; mGAPDH, forward 5′-CGTGCCGCCTGGAGAAACC-3′, reverse 5′-TGGAAGAGTG GGAGTTGCTGTTG-3′.

### Liver histology

Liver samples were fixed in 4% paraformaldehyde for at least 24 h, snap-frozen and embedded in an OCT compound. Eight micron frozen sections were prepared and stained with oil red O.

### ELISA

ELISAs (Proteintech) were conducted according to the manufacturer’s instructions. Briefly, mouse plasma was incubated with assay diluent for 2 h at room temperature. After washing four times, PCSK9 conjugate was added to each well. After incubation for 2 h, wells were washed and substrate solution was then added. After 30 min at room temperature, stop solution was added and incubated for 30 min. The optical density of each well was determined at a wavelength of 450 nm.

### Dil-LDL uptake assay

Huh7 cells were incubated with serum-free minimum essential medium (MEM) containing 1% antibiotic–antimycotic and fluorescent-tagged LDL (Dil-LDL, 30 ng/mL) for 4 h. The medium was then changed to MEM containing 10% fetal bovine serum, 1% antibiotic-antimycotic, and DAPI for 5 min. After washing with PBS and fixing in 4% paraformaldehyde, the fluorescence in cells was photographed and quantified using the PerkinElmer Operetta CLS imaging system.

### Luciferase assay

The human *PCSK9* 3′-UTR containing the putative miR-181d-5p binding site was subcloned into the pMIR-REPORT vector (Ambion) to generate the pMIR-Luc-PCSK9-3′-UTR WT reporter (Luc-PCSK9 (WT)). Then the CGG to GCC mutation in the miR-181d-5p binding seed sequence of the *PCSK9* 3′-UTR was introduced into the Luc-PCSK9 (WT) plasmid using the QuikChange Lightning Multi Site-Directed Mutagenesis Kit (Agilent Technologies). *Renilla* luciferase plasmid (pRL-TK) was used as the transfection control. Luc-PCSK9 (MT), and control pRL-TK plasmids were cotransfected into Huh7 cells using Lipofectamine 3000 (Invitrogen). At 24 h after transfection, cells were lysed and luciferase activity was measured using a Dual-Glo Luciferase Reporter Assay Kit (Promega).

### Statistical analysis

The results of *in vitro* experiments are presented as the mean ± s.d. and the data from *in vivo* experiments are presented as the mean ± s.e.m. Experiments with two groups were compared by the two-tailed Student’s *t*-test for parametric data or the Mann–Whitney *U* test for nonparametric data. Experiments with more than two groups were compared by one-way ANOVA with Bonferroni’s *post hoc* test for parametric data or a Kruskal–Wallis test with Dunn’s multiple comparisons for nonparametric data. All statistical analyses were performed with Prism version 9.0 (GraphPad Software, Inc., USA) (*P < 0.05, **P < 0.01, ***P < 0.001 as indicated).

## Results

### miR-181d-5p expression is decreased in hypercholesterolemia animal models and negatively correlates with LDL-C levels

**Figure 1:**
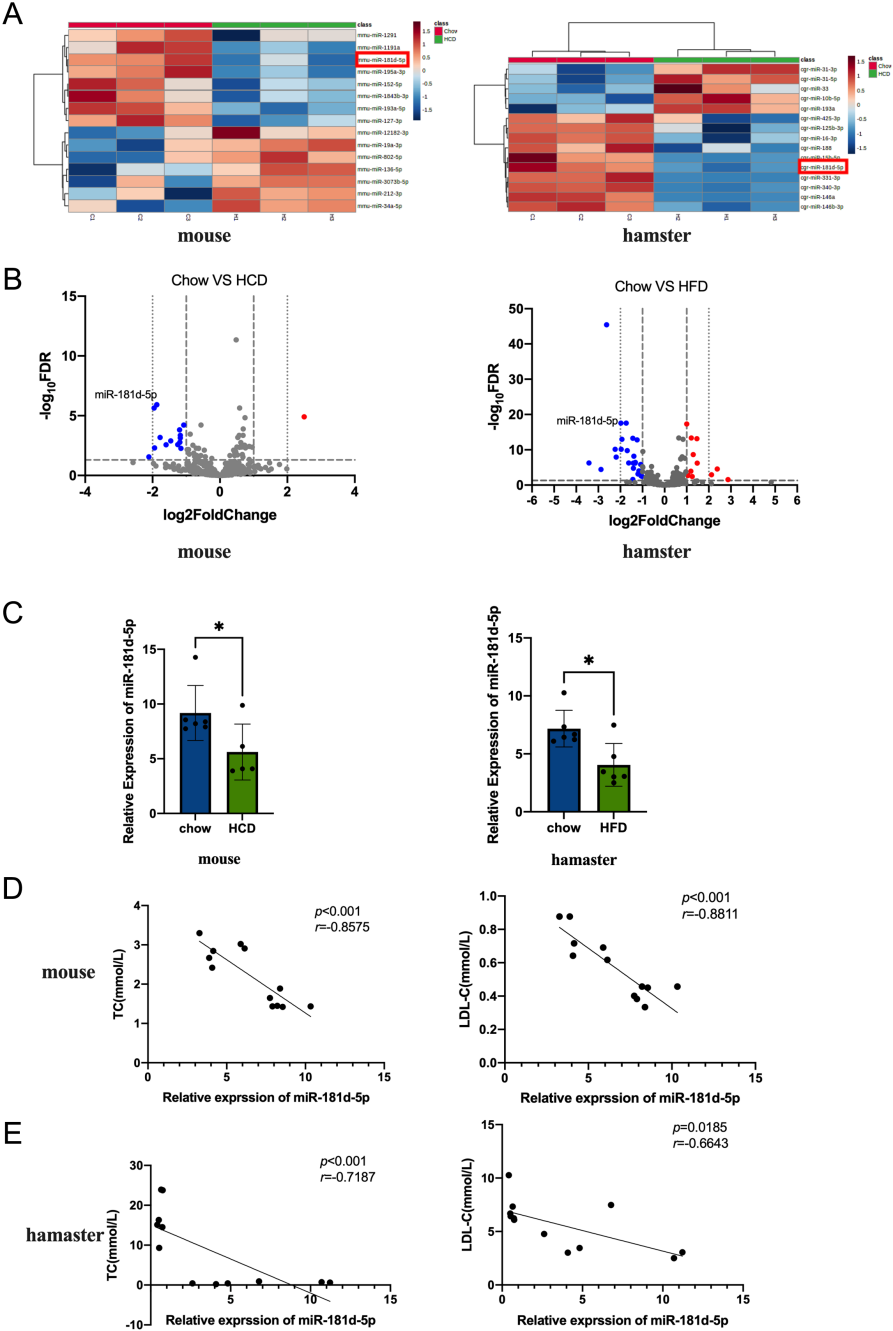
miR-181d-5p levels and their correlation with LDL-C in different hypercholesterolemia animal models. (A) Heatmaps of miRNA expression in the livers of high-cholesterol model mice and hamsters. (B) VIP plots of each miRNA variation in pairwise comparisons of high-cholesterol diet (HCD) and normal chow mouse and hamster groups; the dashed lines indicate the threshold for a significant difference. (C) The expression level of miR-181d-5p in the livers of hypercholesterolemia model mice and hamsters (*n* = 6). (D) Relationships between miR-181d-5p and TC, and miR-181d-5p and LDL-C in hypercholesterolemia mice (*n* = 12) and hamsters (*n* = 12). Statistical significance was calculated using an independent sample *t*-test; *P < 0.05 compared with the chow group. A full colour version of this figure available at https://doi.org/10.1530/JOE-23-0402.

We established hypercholesterolemia mouse and hamster models by feeding the animals high-cholesterol diets (HCDs) (Supplementary Fig. 1A, see the section on supplementary materials given at the end of this article). RNomics analysis of livers from HCD-fed mice and hamsters showed reduced expression of miR-181d-5p (Fig. 1A and B). To further determine the expression of miR-181d-5p in the mouse and hamster hypercholesterolemia models, we used RT-PCR to detect liver miR-181d-5p levels (Fig. 1C). The expression of miR-181d-5p in the livers of the model animals was decreased compared with that in the normal control groups (*P* < 0.05). The serum levels of total cholesterol (TC), LDL-C, and miR-181d-5p in the hypercholesterolemia model animals showed the opposite trend, indicating that miR-181d-5p negatively correlated with the serum levels of TC and LDL-C in the hypercholesterolemia animal models (Fig. 1D and E).

### Overexpression of miR-181d-5p reduces LDL-C levels in hypercholesterolemia model mice

**Figure 2:**
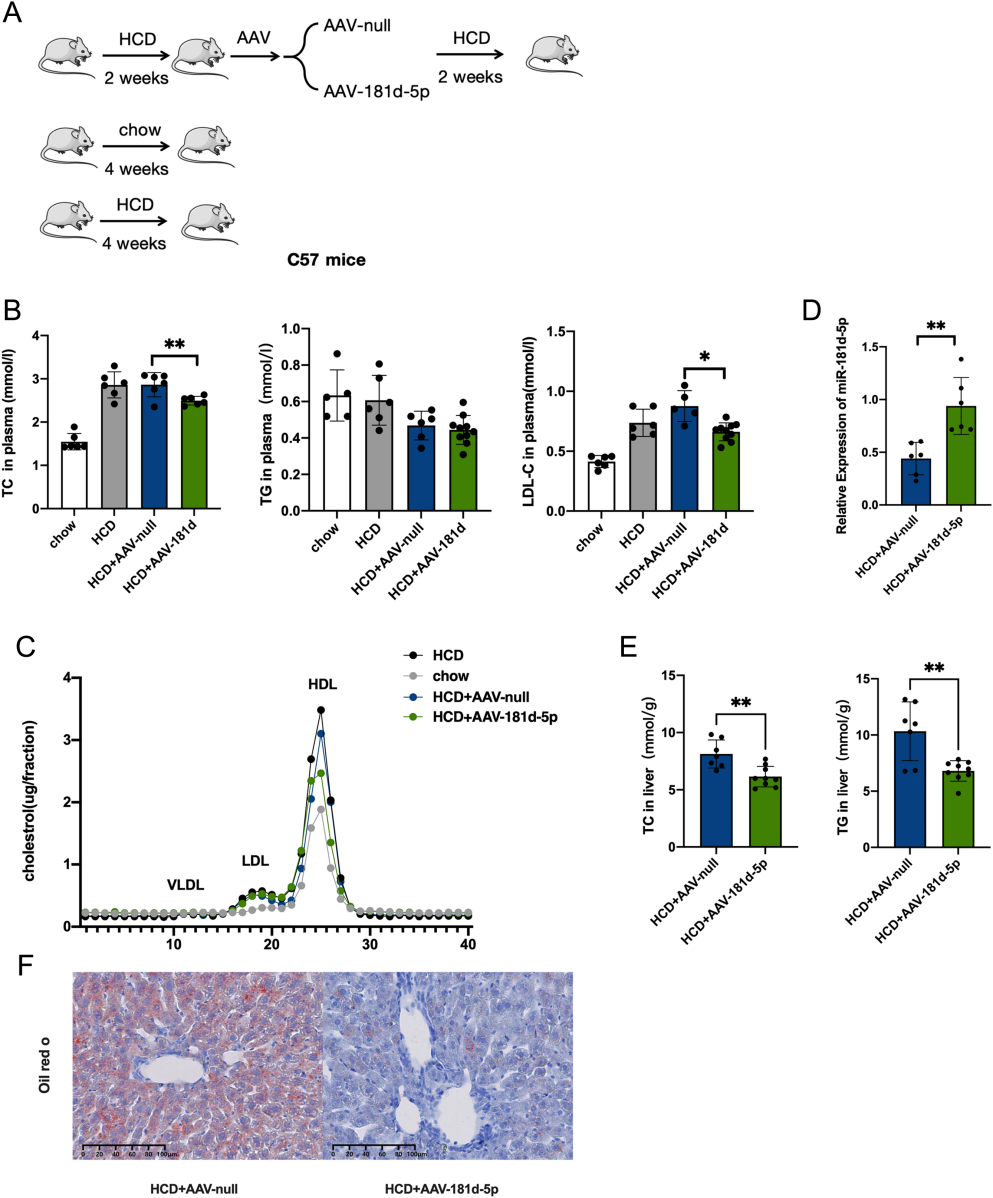
Effects of miR-181d-5p on serum and liver cholesterol in hypercholesterolemia animal models. (A) Schematic depiction of the animal experiments. (B, C) Serum levels of TC, TG, LDL-C (*n* = 6). (D) RT-PCR analysis of the relative miR-181d-5p levels in the miR-181d-5p overexpression and control groups (*n* = 6 per group). (E) TC in the liver and TG in the liver. (F) Lipid deposition in AAV-null- and AAV-181d-5p-treated mice detected by oil red O staining. In (D) and (E), statistical significance was calculated using an independent sample *t*-test; In (B), statistical significance was calculated using one-way ANOVA with a Bonferroni’s *post hoc* test between two indicated groups; *P < 0.05, **P < 0.01, compared with the control group. A full colour version of this figure available at https://doi.org/10.1530/JOE-23-0402.

To further examine the link between miR-181d-5p and LDL-C, AAV-mediated liver-directed miRNA overexpression was used to investigate the effect of miR-181d-5p on LDL-C in the HCD-fed mouse model (Fig. 2A). The overexpression of miR-181d-5p did not affect the serum levels of alanine transaminase (ALT) or aspartate transaminase (AST) (Supplementary Fig. 2A). There were no apparent differences in physical appearance, behavior, food intake, body weight, liver weight, or liver weight ratio (Supplementary Fig. 2B) between the model and control animals. After overexpression of miR-181d-5p for 2 weeks, the levels of hepatic miR-181d-5p and the serum lipid profile were determined. The levels of miR-181d-5p were obviously increased (Fig. 2B and C). In addition, the liver levels of TC and triglyceride (TG) in the miR-181d-5p overexpression group were significantly decreased compared with the control group (Fig. 2E). Moreover, oil red O staining showed reduced lipid deposition in the liver of miR-181d-5p overexpression group mice (Fig. 2F).

### miR-181d-5p targets *PCSK9*

**Figure 3:**
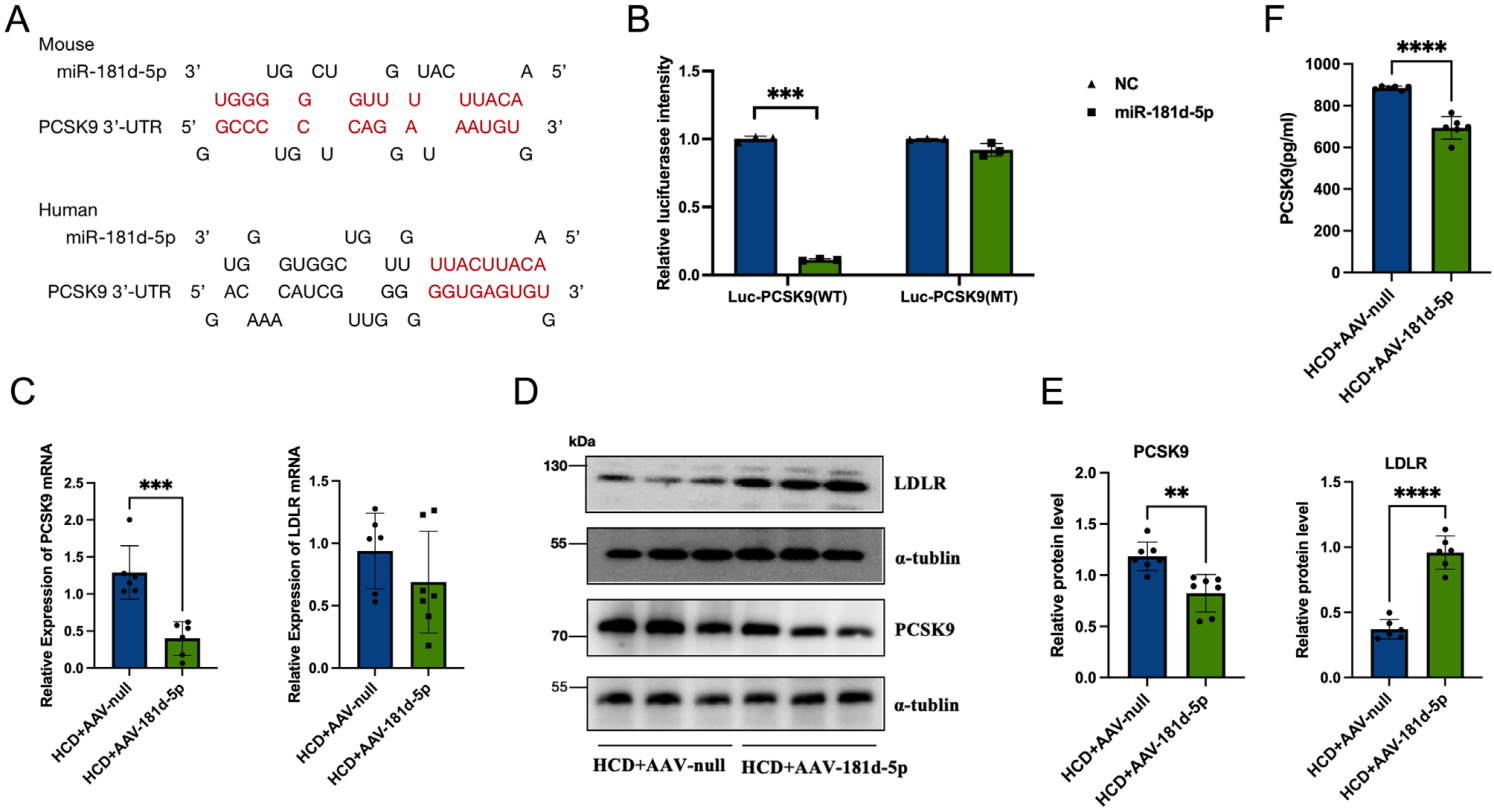
miR-181d-5p targets *PCSK9*. (A) Bioinformatic prediction of miR-181d-5p binding sites in human and mouse *PCSK9* 3′-UTRs. (B) Relative luciferase reporter activity in wild-type and *PCSK9* 3′-UTR binding site mutant cells transfected with miR-181d-5p. (C) RT-PCR was used to detect PCSK9 and LDLR mRNA levels in the livers of AAV-null and AAV-181d-5p groups. (D, E) The protein levels of PCSK9 and LDLR in the livers of AAV-null and AAV-181d-5p groups were detected by western blotting. (F) ELISA analysis of the PCSK9 protein in the plasma of miRNA overexpression and control mice (*n* = 6/each group). Statistical significance was calculated using an independent sample *t*-test; *P < 0.05, **P < 0.01, ***P < 0.001 compared with the AAV-null group. A full colour version of this figure available at https://doi.org/10.1530/JOE-23-0402.

To determine the underlying molecular mechanism by which miR-181d-5p reduces cholesterol levels, we used TargetScan8.0 and miRWALK to predict possible target genes regulated by miR-181d-5p. This analysis identified *PCSK9*, which is associated with the LDL-C clearance pathway. The mechanism of miRNA-mediated gene regulation is mainly post-transcriptional inhibition through binding to the 3′-UTR of target mRNAs. Bioinformatic methods were used to locate 3′-UTR binding sites of miR-181d-5p in humans and mice (Fig. 3A). To confirm whether miR-181d-5p targets the predicted *PCSK9* 3′-UTR binding site, luciferase reporter plasmids containing a mutated 3′-UTR miR-181d-5p binding site in human *PCSK9* and the wild-type *PCSK9* 3′-UTR were constructed. As expected, miR-181d-5p significantly reduced wild-type *PCSK9* 3′-UTR luciferase reporter plasmid activity and had no effect on mutant *PCSK9* 3′-UTR binding site luciferase reporter plasmid activity (Fig. 3B). *PCSK9* mRNA was detected by RT-PCR in the miR-181d-5p overexpression and control groups (Fig. 3C). Meanwhile, compared with the control group, the protein level of PCSK9 was decreased in the miR-181d-5p overexpression group (Fig. 3D and E). PCSK9 can promote the degradation of the low-density lipoprotein receptor (LDLR) and inhibit the reabsorption of LDL, thereby affecting serum LDL-C levels (Zhang *et al.* 2007). Therefore, we determined the levels of LDLR mRNA and LDLR protein in the livers of miR-181d-5p overexpression and control group mice. The LDLR mRNA level in the miR-181d-5p overexpression group was unchanged compared with the control group, while the LDLR protein level was significantly higher than that of the control group (Fig. 3C–E).

### miR-181d-5p increases LDL-C uptake in hepatocytes by inhibiting PCSK9 expression

**Figure 4:**
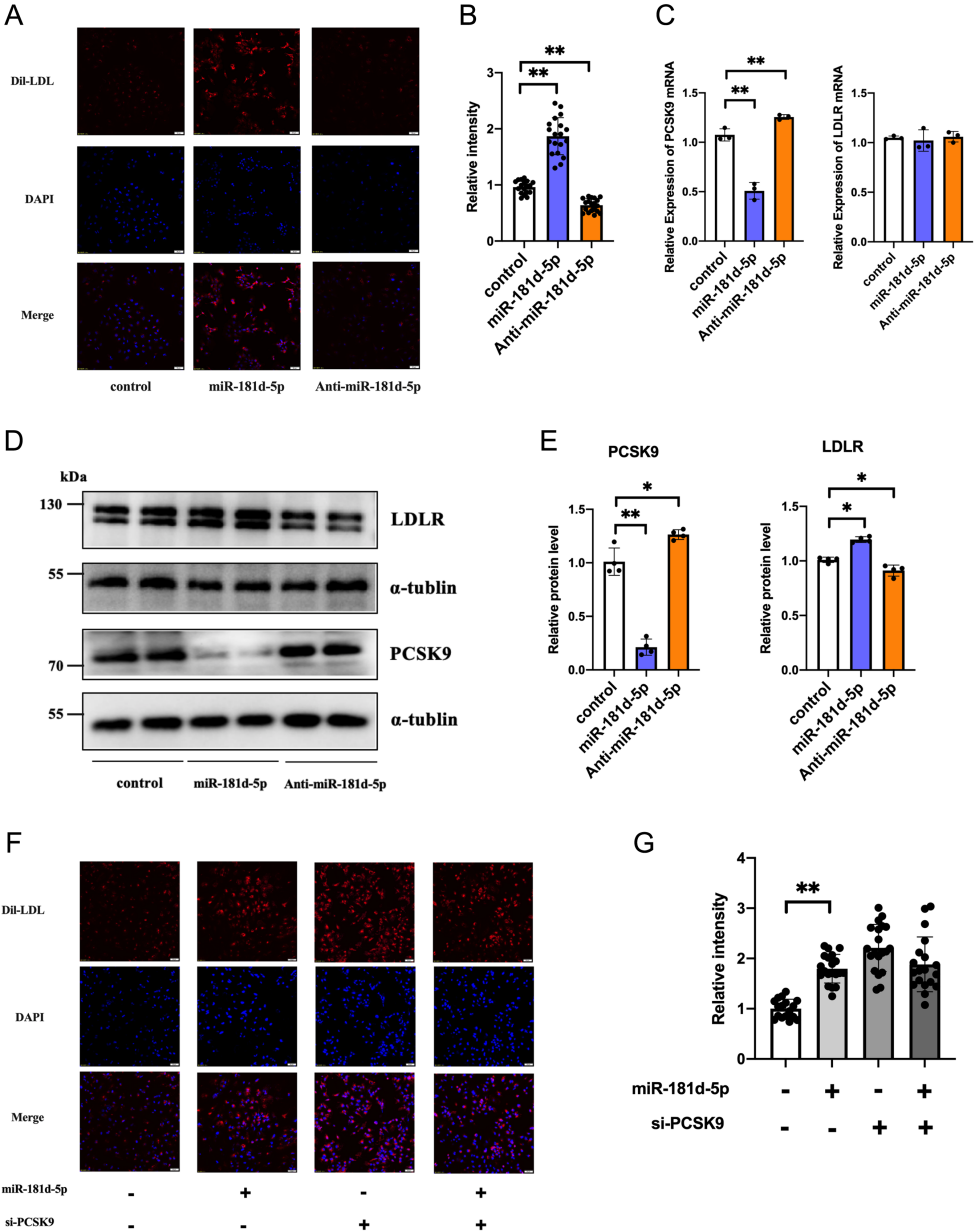
miR-181d-5p increases LDL-C uptake by inhibiting *PCSK9* expression. (A) Dil-LDL fluorescence staining of Huh7 cells after transfection with miR-181d-5p and anti-miR-181d-5p. (B) Quantification of the fluorescence in (A). (C) RT-PCR analysis of *PCSK9* and LDLR mRNAs in Huh7 cells. (D, E) Western blot detection of PCSK9 and LDLR protein levels in Huh7 cells. (F) Dil-LDL (red) and DAPI (blue) fluorescence after co-transfection of Huh7 cells with miR-181d-5p or its control and *PCSK9* siRNA. (G) Quantification of the fluorescence in (F). Statistical significance was calculated using one-way ANOVA with a Bonferroni’s *post hoc* test between two indicated groups; *P < 0.05, **P < 0.01, compared with the control group. A full colour version of this figure available at https://doi.org/10.1530/JOE-23-0402.

PCSK9 can destroy LDLR on the surface of hepatocytes, thereby regulating plasma LDL-C levels (Seidah & Prat 2022). To demonstrate its influence on the cell’s ability to take up LDL-C, we tested the effects of miR-181d-5p on LDLR protein and LDL-C uptake capacity in Huh7 cells. We assessed the impact of miR-181d-5p on LDLR function using a Dil-LDL uptake experiment, where the fluorescence intensity in Huh7 cells indicated their LDL-C uptake capacity. As shown in Fig. 4A, miR-181d-5p significantly enhanced the uptake of LDL-C. Conversely, transfection with anti-miR-181d-5p to inhibit miR-181d-5p reduced LDL-C uptake in Huh7 cells as expected. Overexpression of miR-181d-5p inhibited PCSK9 protein expression while up-regulating LDLR protein expression. In contrast, inhibition of miR-181d-5p decreased LDLR protein expression (Fig. 4C–E). As known, PCSK9 is not the sole regulator of LDL-C uptake (Luo *et al.* 2020). We silenced the basal expression of PCSK9 in Huh7 cells using si-PCSK9. Upon co-transfection with miR-181d-5p or NC, miR-181d-5p failed to inhibit LDL-C uptake following PCSK9 interference (Fig. 4F–G).

**Figure 5:**
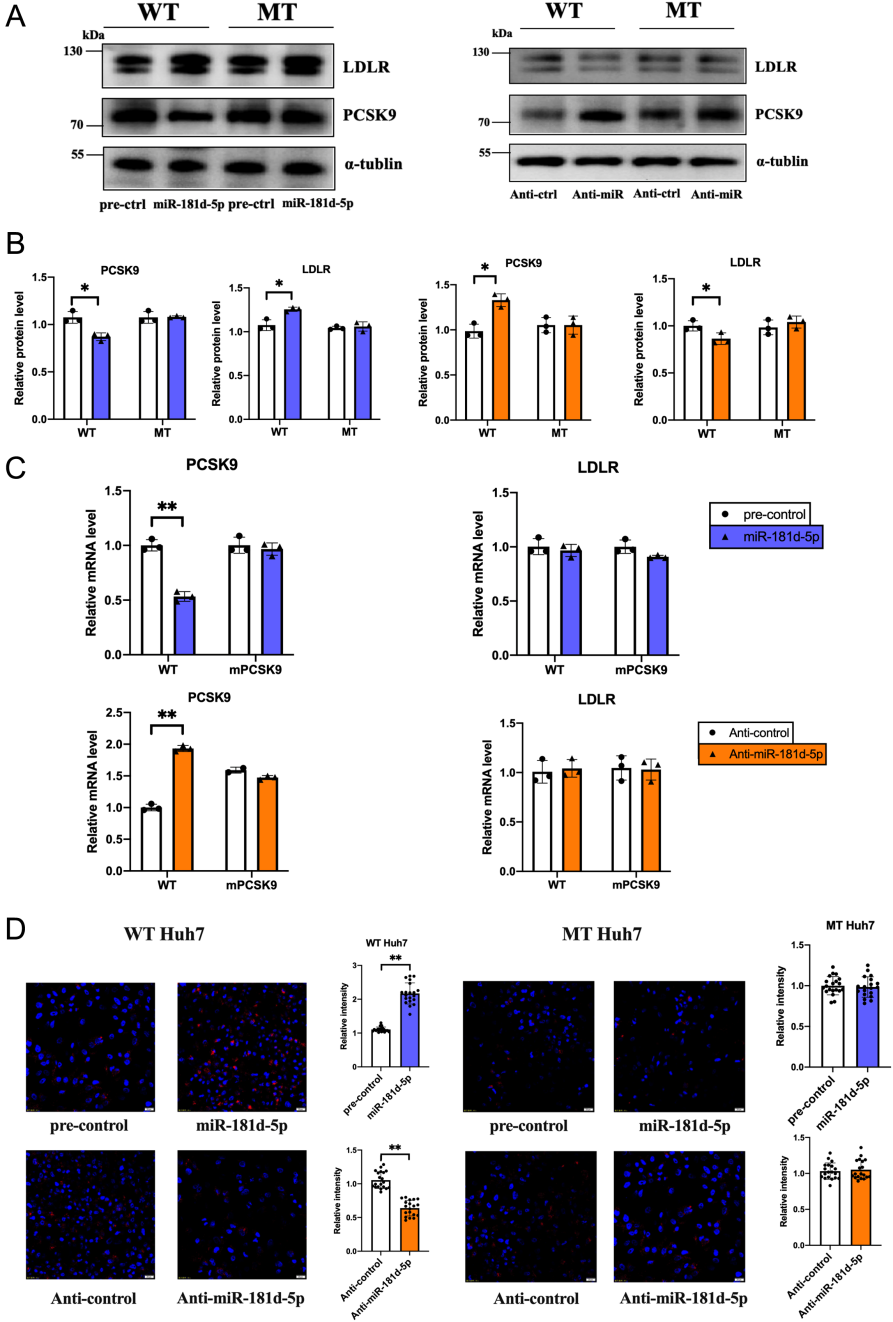
Overexpression of miR-181d-5p increases LDL-C uptake by targeting the predicted *PCSK9* binding site. Wild-type Huh7 and MT Huh7 cells were transfected with miR-181d-5p or anti-miR-181d-5p as indicated. (A, B) Western blot detection of PCSK9 and LDLR protein levels in wild-type and MT Huh7 cells. (C) RT-PCR analysis of *PCSK9* and LDLR mRNA in wild-type and MT Huh7 cells. (D) Dil-LDL fluorescence after transfection with miR-181d-5p or anti-miR-181d-5p. A full colour version of this figure available at https://doi.org/10.1530/JOE-23-0402.

To further confirm miR-181d-5p targeting of *PCSK9*, we used CRISPR/Cas9 gene editing to create a lentivirus containing an miR-181d-5p target site-deleted *PCSK9* 3′-UTR (Supplementary Fig. 3). Transfection of Huh7 cells with this lentivirus (MT Huh7 cells) or with anti–miR-181d-5p did not significantly change the mRNA or protein levels of PCSK9 or LDLR compared with untreated cells (Fig. 5A–C).

Overexpression of miR-181d-5p in Huh7 cells significantly increased the absorption of fluorescence-labeled LDL. The miR-181d-5p-mediated increase in LDL absorption was absent in MT Huh7 cells. Transfection of anti–miR-181d-5p had the opposite effect and decreased LDL absorption in Huh7 cells but did not impact LDL binding in MT Huh7 cells (Fig. 5D).

### Overexpression of miR-181d-5p has no effect on the plasma cholesterol level in LDLR knockout mice

**Figure 6:**
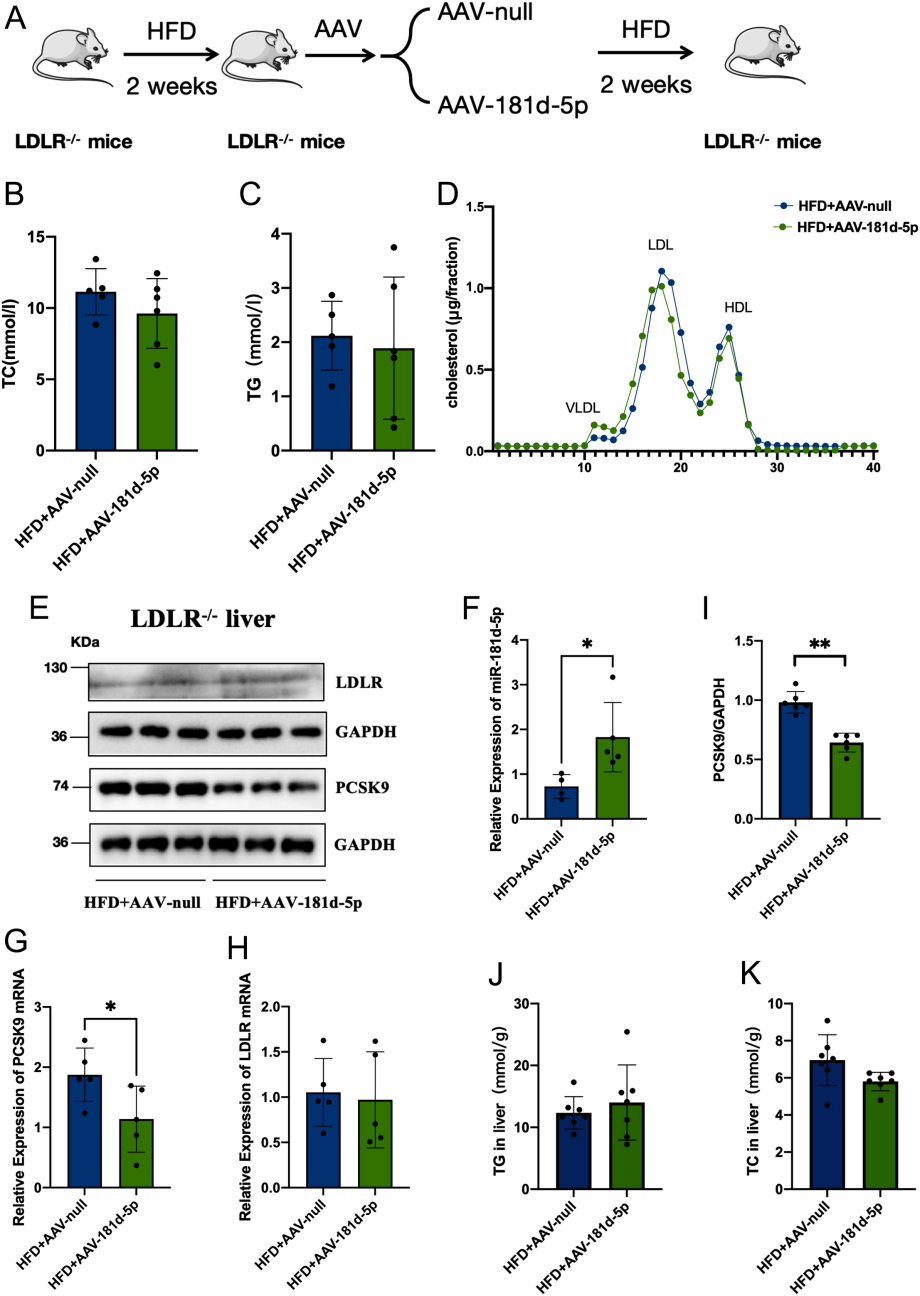
Overexpression of miR-181d-5p does not reduce cholesterol levels in LDLR knockout mice. (A) Schematic depiction of the animal experiments. (B–D) Serum levels of TC and TG (*n* = 6). (E, F) Western blot analysis of PCSK9 and LDLR protein levels in control and miRNA-181d-5p groups. (G, H) RT-PCR detection of *PCSK9* and LDLR mRNA levels in the livers of AAV-null and AAV-181d-5p groups. (I) RT-PCR analysis of the relative miR-181d-5p levels in the miRNA overexpression and control groups. Statistical significance was calculated using an independent sample t-test; *P < 0.05, **P < 0.01, compared with the control group. A full colour version of this figure available at https://doi.org/10.1530/JOE-23-0402.

To examine whether miR-181d-5p can regulate LDL-C *in vivo* by targeting pathways other than the PCSK9/LDLR pathway, we overexpressed miR-181d-5p in LDLR knockout (LDLR^−/−^) mice (Fig. 6A). After 2 weeks of miR-181d-5p overexpression, serum TC and TG levels remained unchanged (Fig. 6B–D) and PCSK9 protein and mRNA levels in the liver were decreased (Fig. 6E–H). In addition, we also measured TC and TG levels in liver tissue. Compared with the control group, the levels of TC and TG in liver tissue of the miR-181d-5p overexpression group remained unchanged (Fig. 6J and K). No apparent differences in physical appearance, behavior, body weight, and food intake were observed in LDLR^−/−^ mice (Supplementary Fig. 4).

## Discussion

Treatment to lower LDL-C levels may benefit patients at high risk of ASCVD (Borén *et al.* 2020). This study found that the expression of miR-181d-5p was increased in the livers of hypercholesterolemia model mice and hamsters. Moreover, liver miR-181d-5p levels negatively correlated with serum LDL-C levels. Exogenous administration of miR-181d-5p significantly reduced total cholesterol levels and LDL-C in hypercholesterolemia model mice. This occurred through miR-181d-5p inhibiting the expression of *PCSK9*, thereby increasing the expression of LDLR in hepatocytes. The binding site between miR-181d-5p and the 3′-UTR of *PCSK9* mRNA was bioinformatically predicted and verified by a Luc-*PCSK9* reporter gene assay. miR-181d-5p targeting of the *PCSK9*-3′-UTR was validated by comparing the mRNA and protein levels of PCSK9 and LDLR in Huh7 cells transfected with wild-type *PCSK9*-3′-UTR and binding site mutant *PCSK9*-3′-UTR lentiviruses. miR-181d-5p targeting of the *PCSK9*-3′-UTR was also verified *in vivo* by transfecting mice with wild-type AAV-3′-UTR or mutant AAV-3′-UTR and measuring circulating LDL-C and liver PCSK9 levels. In addition to miR-181d-5p, miR-222, miR-191, miR-224, and miR-483 can bind directly to the *PCSK9* 3′-UTR to inhibit its expression.

PCSK9 belongs to the proprotein convertase family (Seidah 2021). It is involved in plasma lipid regulation by binding to the LDLR on the surface of liver cells, causing LDLR degradation (Gong *et al.* 2017). In recent years, PCSK9 has received more and more attention as a therapeutic target for hyperlipidemia. Two PCSK9 inhibitors currently on the market, evolocumab and alirocumab, have been used to treat patients with familial hypercholesterolemia (Fitzgerald & Kiernan 2018). In addition, the *PCSK9* siRNA drug, inclisiran, has been successively approved by the European Medicines Agency (EMA) and the U.S. Food and Drug Administration for the reduction of cholesterol levels in patients with primary hypercholesterolemia or mixed dyslipidemia, heterozygous familial hypercholesterolemia, and atherosclerotic cardiovascular disease (Dec *et al.* 2023). Inclisiran can be used in combination with statins or other lipid-lowering therapies to increase the lipid-lowering effect (Frampton 2023). These positive results also demonstrate the potential of PCSK9 gene inhibitors. PCSK9 inhibitors can increase antiviral interferon levels, thereby benefiting dengue patients. Inhibition of PCSK9 may also promote intra-tumor T-cell infiltration, thereby making tumors more responsive to immune checkpoint therapy (Liu *et al.* 2020). Drugs targeting miR-181d-5p, an inhibitor of PCSK9, may therefore have broad application in the treatment of hyperlipidemia, viral infection, and cancer.

miR-181d-5p can inhibit cancer cell proliferation, migration, and invasion in various cancers (Chen *et al.* 2018, Dong *et al.* 2021). In addition, miR-181d can reduce levels of triglycerides and cholesterol esters in hepatocytes (Whittaker *et al.* 2010), and the expression of miR-181d is decreased in plasma and adipose tissue of obese people (Abu-Farha *et al.* 2019) indicating that miR-181d plays an essential role in regulating lipid metabolism. miR-181d-5p therefore provides a new drug option as a *PCSK9* inhibitor. In addition, miR-181d can target the *ANGPTL3* 3′-UTR, inhibit *ANGPTL3* expression, and lipoprotein lipase activity (Abu-Farha *et al.* 2019). Therefore, it can play a pleiotropic role.

With the progressive understanding of small nucleic acid molecules, the clinical use of miRNAs as markers and for treatment has advanced (Vienberg *et al.* 2017). miRNAs are involved in the regulation of various diseases and almost all metabolic systems (Agbu & Carthew 2021) and the development of miRNA drugs based on miR-34 and miR-122 has reached phase II clinical trials (Thakral & Ghoshal 2015, Rupaimoole & Slack 2017). Currently, miRNA mimics and anti-miRNA inhibitors (Saliminejad *et al.* 2019) are being developed. Problems in the delivery of miRNAs limit their therapeutic effect. miRNA mimics or inhibitors are easily degraded by circulating RNases. In addition, miRNAs may be absorbed by non-target organs, leading to non-specific effects, while the extracellular matrix can prevent miRNA mimics or inhibitors from entering cells. The negative charge and relatively large molecular mass of nucleic acids also affect their entry into the cytoplasm through the phospholipid bilayer (Ho *et al.* 2022). Improved delivery systems are needed to overcome miRNA delivery barriers to optimize the therapeutic effects of miRNAs. miRNA delivery systems include lipid transport systems, polyethylenimine transport systems, dendritic macromolecules, and polylactide-hydroxyacetate particles (Zhang *et al.* 2013). In summary, our study shows a correlation between miR-181d-5p and LDL-C, and that miR-181d-5p reduces serum LDL-C levels by regulating the expression of *PCSK9*. These results indicate miR-181d-5p to be a new target for lipid reduction.

### Limitations of the study

We only evaluated the effectiveness of miR-181d-5p delivery via AAV but did not evaluate its safety. Other delivery methods need to be further explored in the future. The *PCSK9* binding site for miR-181d was only validated in cells. AAVs for *in vivo* validation should therefore be generated and tested.

## Supplementary Materials

Supplementary Material

## Declaration of interest

The authors declare that there is no conflict of interest that could be perceived as prejudicing the impartiality of the study reported.

## Funding statement

This work was supported by the National Key Research and Development Program of China (grant nos 2021YFC2500600 and 2021YFC2500603), the National Natural Science Foundation of China (grant nos 81970224), and the Beijing Municipal Health Commission (2021-7; 2021-1854).

## Author contributions

YWQ conceived and designed the study. YW and FL prepared the samples and conducted the animal experiments. YW performed the data analysis. YW and FL assisted in the statistical analysis of the metadata. YW and YWQ wrote the manuscript.

## References

[bib1] Abu-Farha M Cherian P Al-Khairi I Nizam R Alkandari A Arefanian H Tuomilehto J Al-Mulla F & Abubaker J 2019 Reduced miR-181d level in obesity and its role in lipid metabolism via regulation of ANGPTL3. Scientific Reports 9 11866. (10.1038/s41598-019-48371-2) 31413305 PMC6694160

[bib2] Agbu P & Carthew RW 2021 MicroRNA-mediated regulation of glucose and lipid metabolism. Nature Reviews. Molecular Cell Biology 22 425–438. (10.1038/s41580-021-00354-w) 33772227 PMC8853826

[bib3] Borén J Chapman MJ Krauss RM Packard CJ Bentzon JF Binder CJ Daemen MJ Demer LL Hegele RA Nicholls SJ*et al.* 2020 Low-density lipoproteins cause atherosclerotic cardiovascular disease: pathophysiological, genetic, and therapeutic insights: a consensus statement from the European Atherosclerosis Society Consensus Panel. European Heart Journal 41 2313–2330. (10.1093/eurheartj/ehz962) 32052833 PMC7308544

[bib4] Chen H Xiao Z Yu R Wang Y Xu R & Zhu X 2018 miR-181d-5p-FOXP1 feedback loop modulates the progression of osteosarcoma. Biochemical and Biophysical Research Communications 503 1434–1441. (10.1016/j.bbrc.2018.07.060) 30031607

[bib5] Citrin KM Fernández-Hernando C & Suárez Y 2021 MicroRNA regulation of cholesterol metabolism. Annals of the New York Academy of Sciences 1495 55–77. (10.1111/nyas.14566) 33521946 PMC8938903

[bib6] Dec A Niemiec A Wojciechowska E Maligłówka M Bułdak Ł Bołdys A & Okopień B 2023 Inclisiran-A revolutionary addition to a cholesterol-lowering therapy. International Journal of Molecular Sciences 24. (10.3390/ijms24076858) PMC10095256 37047830

[bib7] Dong X Liu Y Deng X Shao J Tian S Chen S Huang R Lin Z Chen C & Shen L 2021 C1GALT1, negatively regulated by miR-181d-5p, promotes tumor progression via upregulating RAC1 in lung adenocarcinoma. Frontiers in Cell and Developmental Biology 9 707970. (10.3389/fcell.2021.707970) 34307388 PMC8292976

[bib8] Ference BA Ginsberg HN Graham I Ray KK Packard CJ Bruckert E Hegele RA Krauss RM Raal FJ Schunkert H, *et al.* 2017 Low-density lipoproteins cause atherosclerotic cardiovascular disease. 1. Evidence from genetic, epidemiologic, and clinical studies. A consensus statement from the European Atherosclerosis Society Consensus Panel. European Heart Journal 38 2459–2472. (10.1093/eurheartj/ehx144) 28444290 PMC5837225

[bib9] Fitzgerald G & Kiernan T 2018 PCSK9 inhibitors and LDL reduction: pharmacology, clinical implications, and future perspectives. Expert Review of Cardiovascular Therapy 16 567–578. (10.1080/14779072.2018.1497975) 29979908

[bib10] Frampton JE 2023 Inclisiran: a review in hypercholesterolemia. American Journal of Cardiovascular Drugs 23 219–230. (10.1007/s40256-023-00568-7) 36869996

[bib11] Gong Y Ma Y Ye Z Fu Z Yang P Gao B Guo W Hu D Ye J Ma S, *et al.* 2017 Thyroid stimulating hormone exhibits the impact on LDLR/LDL-c via up-regulating hepatic PCSK9 expression. Metabolism: Clinical and Experimental 76 32–41. (10.1016/j.metabol.2017.07.006) 28987238

[bib12] Ho PTB Clark IM & Le LTT 2022 MicroRNA-based diagnosis and therapy. International Journal of Molecular Sciences 23. (10.3390/ijms23137167) PMC9266664 35806173

[bib13] Janssen HL Reesink HW Lawitz EJ Zeuzem S Rodriguez-Torres M Patel K van der Meer AJ Patick AK Chen A Zhou Y, *et al.* 2013 Treatment of HCV infection by targeting microRNA. New England Journal of Medicine 368 1685–1694. (10.1056/NEJMoa1209026) 23534542

[bib14] Liu X Bao X Hu M Chang H Jiao M Cheng J Xie L Huang Q Li F & Li CY 2020 Inhibition of PCSK9 potentiates immune checkpoint therapy for cancer. Nature 588 693–698. (10.1038/s41586-020-2911-7) 33177715 PMC7770056

[bib29] Luo J Yang H & Song BL 2020 Mechanisms and regulation of cholesterol homeostasis. Nature Reviews Molecular Cell Biology 21 225–245. (10.1038/s41580-019-0190-7) 31848472

[bib16] Mach F Ray KK Wiklund O Corsini A Catapano AL Bruckert E De Backer G Hegele RA Hovingh GK Jacobson TA, *et al.* 2018 Adverse effects of statin therapy: perception vs. the evidence - focus on glucose homeostasis, cognitive, renal and hepatic function, haemorrhagic stroke and cataract. European Heart Journal 39 2526–2539. (10.1093/eurheartj/ehy182) 29718253 PMC6047411

[bib15] Mach F Baigent C Catapano AL Koskinas KC Casula M Badimon L Chapman MJ De Backer GG Delgado V Ference BA, *et al.* 2020 2019 ESC/EAS Guidelines for the management of dyslipidaemias: lipid modification to reduce cardiovascular risk. European Heart Journal 41 111–188. (10.1093/eurheartj/ehz455) 31504418

[bib17] Pirillo A Casula M Olmastroni E Norata GD & Catapano AL 2021 Global epidemiology of dyslipidaemias. Nature Reviews. Cardiology 18 689–700. (10.1038/s41569-021-00541-4) 33833450

[bib18] Rupaimoole R & Slack FJ 2017 MicroRNA therapeutics: towards a new era for the management of cancer and other diseases. Nature Reviews. Drug Discovery 16 203–222. (10.1038/nrd.2016.246) 28209991

[bib19] Saliminejad K Khorram Khorshid HR Soleymani Fard S & Ghaffari SH 2019 An overview of microRNAs: biology, functions, therapeutics, and analysis methods. Journal of Cellular Physiology 234 5451–5465. (10.1002/jcp.27486) 30471116

[bib20] Seidah NG 2021 The PCSK9 discovery, an inactive protease with varied functions in hypercholesterolemia, viral infections, and cancer. Journal of Lipid Research 62 100130. (10.1016/j.jlr.2021.100130) 34606887 PMC8551645

[bib30] Seidah NG & Prat A 2022 The multifaceted biology of PCSK9. Endocrine Reviews 43 558–582. (10.1210/endrev/bnab035) 35552680 PMC9113161

[bib21] Thakral S & Ghoshal K 2015 miR-122 is a unique molecule with great potential in diagnosis, prognosis of liver disease, and therapy both as miRNA mimic and antimir. Current Gene Therapy 15 142–150. (10.2174/1566523214666141224095610) 25537773 PMC4439190

[bib22] Trinder M Francis GA & Brunham LR 2020 Association of monogenic vs polygenic hypercholesterolemia with risk of atherosclerotic cardiovascular disease. JAMA Cardiology 5 390–399. (10.1001/jamacardio.2019.5954) 32049305 PMC7042820

[bib23] Vienberg S Geiger J Madsen S & Dalgaard LT 2017 MicroRNAs in metabolism. Acta Physiologica (Oxford, England) 219 346–361. (10.1111/apha.12681) 27009502 PMC5297868

[bib24] Wang JQ Li LL Hu A Deng G Wei J Li YF Liu YB Lu XY Qiu ZP Shi XJ, *et al.* 2022 Inhibition of ASGR1 decreases lipid levels by promoting cholesterol excretion. Nature 608 413–420. (10.1038/s41586-022-05006-3) 35922515

[bib25] Whittaker R Loy PA Sisman E Suyama E Aza-Blanc P Ingermanson RS Price JH & McDonough PM 2010 Identification of microRNAs that control lipid droplet formation and growth in hepatocytes via high-content screening. Journal of Biomolecular Screening 15 798–805. (10.1177/1087057110374991) 20639500

[bib26] Zhang DW Lagace TA Garuti R Zhao Z McDonald M Horton JD Cohen JC & Hobbs HH 2007 Binding of proprotein convertase subtilisin/kexin type 9 to epidermal growth factor-like repeat A of low density lipoprotein receptor decreases receptor recycling and increases degradation. Journal of Biological Chemistry 282 18602–18612. (10.1074/jbc.M702027200) 17452316

[bib28] Zhang Y Wang Z & Gemeinhart RA 2013 Progress in microRNA delivery. Journal of Controlled Release 172 962–974. (10.1016/j.jconrel.2013.09.015) 24075926 PMC3891846

[bib27] Zhang L Liao Y & Tang L 2019 MicroRNA-34 family: a potential tumor suppressor and therapeutic candidate in cancer. Journal of Experimental and Clinical Cancer Research 38 53. (10.1186/s13046-019-1059-5) 30717802 PMC6360685

