# Supplementary material for: miR-181d-5p ameliorates hypercholesterolemia by targeting PCSK9
Source: J Endocrinol. 2024 Jul 29;262(3):e230402. doi: 10.1530/JOE-23-0402 (PMC11301420; doi:10.1530/JOE-23-0402)
Supplement: Supplementary Material [file supplementary_material.pdf]

Supplementary Information

miR-181d-5p ameliorates hypercholesterolemia by targeting PCSK9

Yu Wang, Fan Li, Xiaoqian Gao, Huahui Yu, Zhiyong Du, Linyi Li, Yanwen Qin*

S1

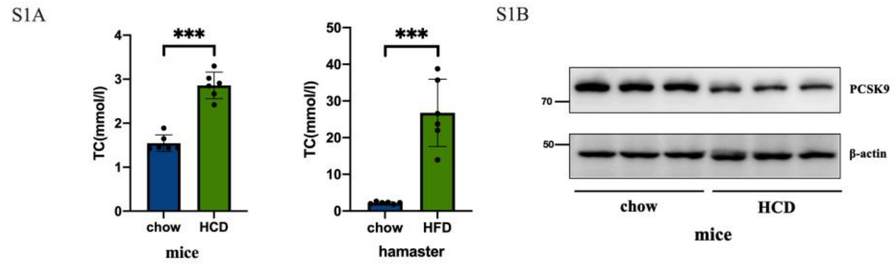

Figure S1. Generation of animal models of hypercholesterolemia. (S1A) C57BL/6J mice (8-week-old) fed a high-cholesterol diet for 2 weeks, had elevated plasma cholesterol levels compared with the control group. Hamsters (6-week-old) fed a high-cholesterol diet for 4 weeks, had elevated plasma cholesterol levels compared with the control group. (S1B) The protein levels of PCSK9 in the livers of chow and HCD groups detected by western blotting. Statistical significance between two indicated groups was calculated using an independent sample t-test; $*p < 0.05$, $**p < 0.01$, compared with the control group.

S2

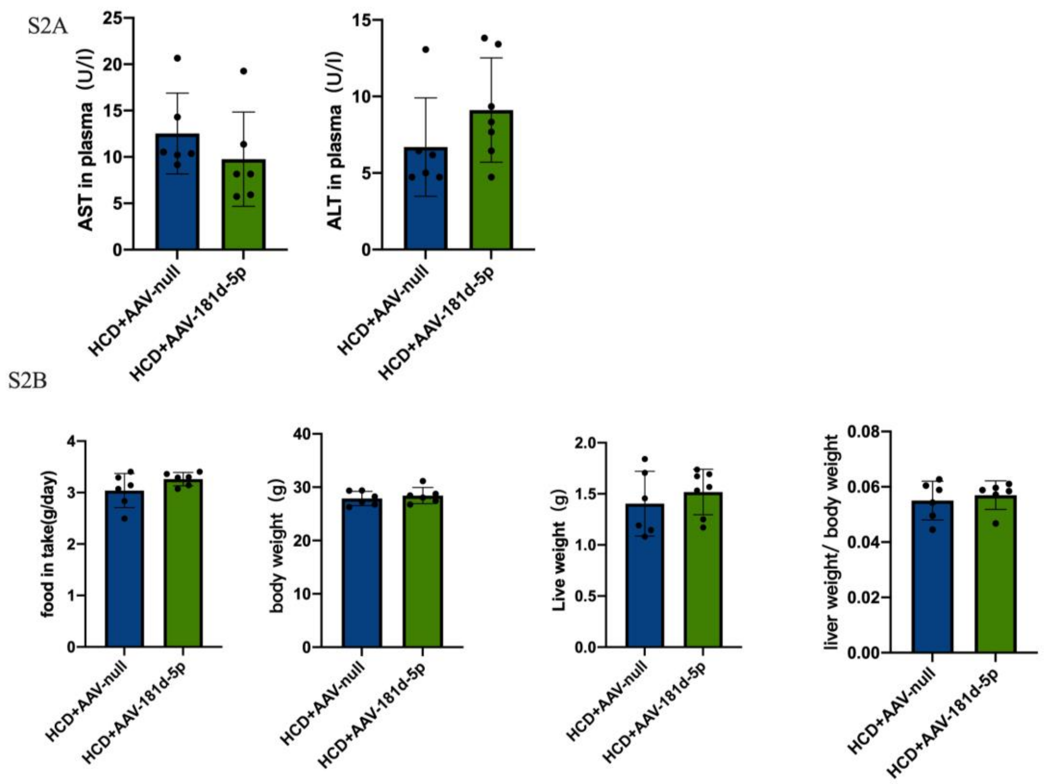

Figure S2. Eight-week-old male C57BL/6J mice fed a high-cholesterol diet for two weeks and then injected with adeno-associated viruses.

(S2A) Mouse plasma AST and ALT levels. (S2B) Food intake, body weight, liver weight, and liver weight in AAV-null and AAV-181d-5p groups.

Statistical significance between two indicated groups was calculated using an independent sample t-test; $*p < 0.05$, $** p < 0.01$, compared with the control group.

S3

Gene name	Genetic species	Gene ID	GenBank ID
PCSK9	Human	255738	NM_174936

sgRNA Number	sgRNA sequence
PCA14414	CGTTGGGGGGTGAGTGTGAA

Carrier name: GV708

Component sequence: U6-sgRNA-EF1a-Cas9-FLAG-CMV-EGFP-P2A-puro

Reference number: CON544

Contrast insertion sequence: CGCTTCCGCGGCCCGTTCAA

Oligo synthesis information

**NO.**	**5’**	**STEM**	**3’**

PCSK9-sgRNA(14414-1)-a	CACCg	CGTTGGGGGGTGAGTGTGAA	
PCSK9-sgRNA(14414-1)-b	aaac	TTCACACTCACCCCCCAACG	c

Figure S3. CRISPR-Cas9 strategy for targeting the miR-181d-5p binding site in the human *PCSK9* 3′-UTR.

S4

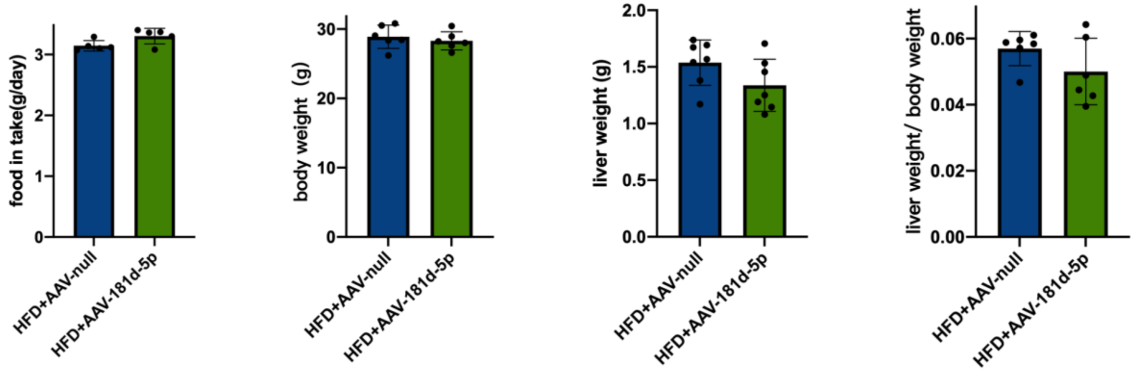

Figure S4. Eight-week-old male *Ldlr*$^{-/-}$ mice fed a high-cholesterol diet for two weeks and then injected with adeno-associated viruses. Food intake, body weight, liver weight, and liver weight to body weight ratio of AAV-null and AAV-181d-5p group mice. Statistical significance between two indicated groups was calculated using an independent sample t-test; *$p < 0.05$, **$p < 0.01$, compared with the control group.
